# Resistance of Soda Residue–Fly Ash Based Geopolymer Mortar to Acid and Sulfate Environments

**DOI:** 10.3390/ma14040785

**Published:** 2021-02-07

**Authors:** Xianhui Zhao, Haoyu Wang, Boyu Zhou, Han Gao, Yonghui Lin

**Affiliations:** 1 School of Civil Engineering, Hebei University of Engineering, Handan 056038, China; 201411601006@stu.hebut.edu.cn; 2 Tianjin University Renai College, Tianjin 301636, China; gaohan@tjrac.edu.cn; 3 School of Civil and Transportation Engineering, Hebei University of Technology, Tianjin 300401, China; 201911601009@stu.hebut.edu.cn (B.Z.); 201611601012@stu.hebut.edu.cn (Y.L.); 4 Department of Economics and Management, Hebei Normal University for Nationalities, Chengde 067000, China

**Keywords:** geopolymer, fly ash, soda residue, chemical attack, compressive strength, microstructure

## Abstract

The early mechanical performances of low-calcium fly ash (FFA)-based geopolymer (FFA–GEO) mortar can be enhanced by soda residue (SR). However, the resistance of SR–FFA–GEO mortar to acid or sulfate environments is unclear, owing to the various inorganic calcium salts in SR. The aim of this study was to investigate the long-term mechanical strengths of up to 360 d and evaluate the resistance of SR–FFA–GEO mortar to 5% HCl and 5% Na_2_SO_4_ environments through the losses in compressive strength and mass. Scanning Electron Microscopy (SEM), Energy-Dispersive Spectroscopy (EDS) and Fourier Transform Infrared Spectrometer (FTIR) experiments were conducted for the SR–FFA–GEO mortars, both before and after chemical attack, to clarify the attack mechanism. The results show that the resistances of the SR–FFA–GEO mortar with 20% SR (namely M10) to 5% HCl and 5% Na_2_SO_4_ environments are superior to those of cement mortar. The environmental HCl reacts with the calcites in SR to produce CaCl_2_, CO_2_ and H_2_O to form more pores under HCl attack, and the environmental Na^+^ cations from Na_2_SO_4_ go into Si-O-Al network structure, to further enhance the strength of mortar under Na_2_SO_4_ attack. These results provide the experimental basis for the durability optimization of SR–FFA–GEO mortars.

## 1. Introduction

In recent years, geopolymer material has obtained rapid development and wide interest in the industry of building material [1,2]. As the sustainable alternatives to conventional cement, geopolymer material has the potential for reducing energy consumption and CO_2_ emission by approximately 60% and 80%, respectively [3,4,5]. As a kind of alkali-activated aluminosilicates, geopolymer material possesses similar (or superior) mechanical properties to Ordinary Portland Cement (OPC)-based material, as well as durable performances, such as high compressive strength, high compatibility with organic components [6], high hardness, high thermal-stability, high fire-resistance, high chemical erosion-resistance, etc. [7,8,9,10]. Currently, some industrial solid wastes composed of aluminosilicate phases, such as fly ash (FA), blast furnace slag, red mud, etc., can be employed for preparing geopolymer products by alkali-activation [8,11,12]. In particular, the reutilization of available industrial solid wastes is of great significance to the international ecological environment.

Low-calcium fly ash (FFA), as one of most commonly used raw materials and most available solid wastes for geopolymer preparation, mainly consists of amorphous spherical beads with aluminosilicate compositions [2,13,14,15]. The amorphous geopolymer can be manufactured with FFA and basic alkaline solution with Na^+^ or K^+^ cations, such as NaOH, KOH, Na_2_SiO_3_ or other mixed solutions by geopolymerization process [13,16,17]. Moreover, the sodium-aluminosilicate-polymer gel (N-A-S-H) has been verified through a series of micro-structural detection experiments [18,19]. Moreover, evenly applied N-A-S-H gel with strong chemical bond energy of Si-O-Al chains makes geopolymer material possess good mechanical strength and superior durability [2,12,18]. However, the FFAs are activated by alkaline solutions, at room or ambient temperatures, due to the incomplete dissolution, as well as the slow hardening rate, presenting the disadvantages of longer setting time, along with slower strength development, and evenly performing that the unconfined compressive strengths at a 28-day age are lower than 10 MPa [17,20,21]. Additionally, Zhao et al. [22] prepared the fly ash–geopolymer cement incorporating soda residue by press-form method. It was revealed that the alkali-activated geopolymer was synthesized from fly ash and soda residue (mass ratio is 1:1) activated by 5% NaOH, which obtains the compressive strength of 22.04 MPa. In particular, the FFA–GEO materials are limited to the precast structure members [23,24] and the backfill materials [22,25]. However, the previous studies have presented that additional calcium source and temperature-curing are two relatively effective external ways to improve the fresh and mechanical performances of FFA–GEO materials [25,26,27,28]. The long pre-curing at room temperature (25 °C) is beneficial for the compressive strength enhancement of FFA–GEO before cured at elevated temperature [19]. For the long-term mechanical strength, FFA–GEO was recommended to cure at room temperature, with sealed treatment to obtain higher compressive strength of up to 180 d [27]. On the other hand, some calcium-additives, such as CaO, Ca(OH)_2_ and CaCO_3_, produce the positive influence on the mechanical properties and the microstructure of FFA–GEO material [29,30,31].

Soda residue (SR), as one of most available chemical industry solid wastes in China, is mainly composed of various inorganic calcium salts—64% CaCO_3_, 10% Ca(OH)_2_, 6% CaCl_2_ and 3% CaSO_4_ [27]. The SRs are the by-product of the industrial Na_2_CO_3_ process, and 7.8~10.0 million tons of SRs are produced annually by more than 50 enterprises in China [32,33]. To achieve the recycling reutilization of the SR, the SR was used as a calcium additive in alkali-activated FFA materials, to prepare geopolymer at room or ambient temperature [22,25,27,28].

In the previous research, it was revealed that the press-formed preparation of alkali-activated fly ash cement reusing the SR (particle size < 2.0 mm), the FFA and 5.0% NaOH mixed with 0.518 liquid–solid ratio (L/S) was feasible as the brick or soil materials [22]. The compressive strength of the designed sample (with the extremely low water absorption of 2.65%) reached 22.04 MPa at 90 d. Owing to the addition of the SR, the final gel products were the mixtures of geopolymeric gels (N,C)-A-S-H and hydrated gels (C-A-S-H and/or C-S-H) at 90 d, by the detections of SEM–EDS, XRD and FTIR tests. Moreover, the SR was reutilized as a calcium source in FFA–GEO as the goaf backfill material alternative to cement-based material [25]. Furthermore, it was feasible for the SR to improve and enhance the fresh and mechanical performances of FFA–GEO backfill pastes at room temperature. The optimal paste SFN6 (SR–FA ratio 2:3, solution concentration 2.0 mol/L and L/S 1.2) obtained high fluidity (260.0 mm), better compressive strength (3.70 MPa), as well as high stone ratio (98.6%) for grouting backfill. The gel products of SFN6 were clarified as the mixtures of (N,C)-A-S-H and C-S-H gels. In addition, the geopolymer cement mortars were prepared by casting method, using SR, FFA and NaOH solution as the alternative to cement products, due to the superior mechanical strength, micro-characteristic and thermal stability [27]. The flexural and compressive strengths of prepared mortars all increased with the additional content of the SR from 0 to 120 g at 60 days old. The curing time of up to 180 d was investigated, and it was found that SR just played a role in the enhancement of early term compressive strength. However, the long-term mechanical performance of up to 360 d is still unclear for soda residue–low-calcium-fly-ash-based geopolymer (SR–FFA–GEO). It is conducive to evaluate the stability and durability of SR–FFA–GEO cement material at long curing ages.

Additionally, the durability and stability of SR–FFA–GEO material are closely related with the gel products and microstructures [27,28]. Moreover, the gel product compositions are determined by the additional contents of calcium sources for alkali-activated FFA materials [26]. The additional calcium-source makes FFA–GEO form (N,C)-A-S-H or the coexistence of C-S-H and N-A-S-H, but the main gel products will be C-S-H and C-A-S-H with the excessive calcium-additives [29]. In particular, the mechanical performances and micro-characteristics were investigated for SR–FFA–GEO cement as building material in the previous study [27], and however, the resistance of SR–FFA–GEO material to chemical attack is still unclear owing to the various inorganic calcium-containing salts. In the aggressive environments of mining, chemical, mineral processing and other industries, the acid and sulfate attack are the desirable performances for the building materials [34]. Therefore, the resistances of SR–FFA–GEO material to acid and sulfate environments should be further explored to supplement the durability of geopolymer materials, guide the optimization in mixing proportion for the larger scale and promote the wider utilization of cleaner productions.

The main aim of this research is to investigate the long-term strength developments of SR–FFA–GEO mortar cured for up to 360 d, and then evaluate the resistances of SR–FFA–GEO mortar to 5% hydrochloric acid (HCl) and 5% sodium sulfate (Na_2_SO_4_) environments through compressive strength loss and mass loss. Furthermore, the reaction mechanisms of HCl and Na_2_SO_4_ attack were investigated through the detections of SEM, EDS and FTIR tests. The results will provide the references for the properties’ optimization and the wider reuse of the SR and the FFA to a larger scale.

## 2. Materials and Methods

### 2.1. Raw Materials

The raw materials for preparing geopolymer (GEO) mortars are low-calcium-fly-ash (FFA) powder (Powder Company, Nanning, China), soda-residue (SR) powder (Sanyou Company, Tangshan, China), fine-aggregate (standard sand) and alkali-activator (sodium hydroxide solution) (Kemiou Company, Tianjin, China). The basic chemical components of FFA and SR are presented by XRF in Table 1, and the physical indexes of the FFA and the SR are shown in Table 2. Moreover, the SEM images and the XRD patterns of raw materials FFA and SR are referenced in the previous studies [22,25,27].

The low-calcium fly ash (FFA) includes 5.32% of active CaO, which is supported by a power plant in Jiangxi Province, Southern China. From the SEM images, the FFA mainly consists of the micro-beads with the powerful potential-reactivity that have a slightly smooth surface, as described by Zhao et al. [22,25,27]. For the XRD pattern, the FFA is essentially composed of 25.32% Al_2_O_3_ and 51.20% SiO_2_; the crystal components of FFA contain mullite and quartz, while the “hump” of amorphous phases (vitreous) is in the range from 19° 2θ to 29° 2θ in the XRD pattern [27].

The soda residue (SR) includes nearly 83% of the inorganic calcium-containing salts -CaCO_3_, CaCl_2_ and Ca(OH)_2_, as well as CaSO_4_, which is derived from a Na_2_CO_3_-production plant in Hebei province, Northern China [22,25,27]. From the SEM picture, the SR possesses porous and loose microstructural morphology, as described by Zhao et al. [27]. From the XRD pattern, the SR consists of halite (NaCl), gypsum (CaSO_4_) and calcite (CaCO_3_) crystal phases. After dried at 40 °C, the SR was first milled and then sieved for 0.5 mm (the particle size ≤ 0.5 mm, and amount passing #325 sieve is 24%), for sample preparation.

Standard stand meets the standards of GB/T 17671-1999 (ISO) [35]. The square hole lengths to the cumulative screening-quality percentage are exactly identical with that used in the previous study [27]. Moreover, the loss on ignition (LOI) is less than 0.40%, SiO_2_ content is high than 98%, as well as mud content (including soluble salts) is less than 0.20%.

Alkali-activator: The sodium hydroxide (NaOH) solution was prepared by dissolving NaOH pellets (purity ≥ 98% and analytical grade) into the distilled water. In general, the 8~14 mol/L molarity concentration of NaOH solution is used to prepare geopolymer samples for the better alkali-activated effects [18,36]. Moreover, considering the economic benefits, in this study, NaOH solution with 8 mol/L concentration was utilized as the alkaline activator. The alkali-activator was cooled to 25 ± 2 °C before sample preparation [27].

Acid and sulfate attack solutions: The 5% hydrochloric acid (HCl) and 5% sodium sulfate (Na_2_SO_4_) solutions were selected to conduct the acid and sulfate attack experiments, for the comparison with the data in References [26,37]. All of the utilized chemical solutions were derived from Baishi company in Tianjin, China.

### 2.2. Preparation of Geopolymer Mortars

The SR–FFA–GEO geopolymer mortars were prepared, using SR powder, FFA powder and NaOH solution. The SR and FFA are used as the precursors for preparing geopolymer. In this work, ten groups of geopolymer mortars were manufactured with the liquid–solid ratio (L/S) of 0.75, as shown in Table 3. Here, L/S means the mass ratio of alkali-activator NaOH solution to solid powders (SR and FFA), and cement–sand ratio (C/S) means the mass ratio of solid powders to standard sands. The costs of each group are identical due to the same L/S and the identical contents of the solid powders.

The solid powders (both of SR and FFA) were firstly blended for three minutes, and then 8 mol/L NaOH solution (25.10% NaOH + 74.90% H_2_O) was mixed with solid powders and stirred for another five minutes, according to Reference [27]. Thereafter, standard sand was added, and then evenly stirred for another ten minutes, to get the fresh mixtures. After the stirring, the fresh mixtures were casted into the prismatic steel molds (the size of 40 mm × 40 mm × 160 mm) by two layers, according to the Chinese standard GB/T 17671-1999 (ISO) [35]. Each layer of the fresh mixtures was vibrated for another two minutes on the vibrating table. In particular, the size of steel molds 40 mm × 40 mm × 160 mm was selected to compare the testing results with that in the previous literature [27,37].

To research the influence of long curing-age up to 360 d on the mechanical strength development of mortar samples, the geopolymer mortar samples were manufactured with the different SR contents (Table 3). Here, the mortar samples were designed to cure respectively for 90, 150 and 360 d, at room temperature (25 ± 3 °C), after being sealed with the plastic films; the curing method of was recommended in the previous study [27]. The prepared mortar samples were demolded after being cured for 60 d, due to the slow hardening at room temperature [38]. Based on the hardening effect, the selection of 60 days to demold is also to reduce the influence of steel mold on the late shrinkage of the geopolymer mortar. After demolded, the original curing condition was continued until the required ages.

### 2.3. Testing Methods

#### 2.3.1. Determination of Fresh and Physical Properties

**Figure 1:**
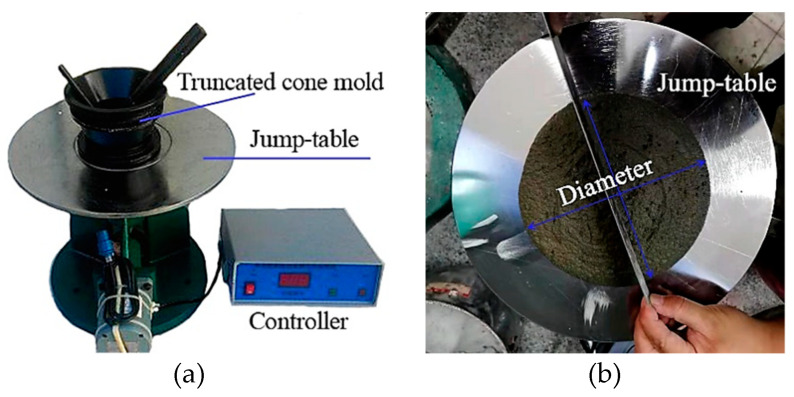
The testing method to the fluidity of SR–FFA based geopolymer mortar: (**a**) Testing device of cement mortar and (**b**) measurement of result.

Fluidity, bulk density, porosity and shrinkage behavior of geopolymer mortars are of significance to characterize the fresh and physical properties. The fluidities of designed fresh mixtures were measured by a fluidity tester for NLD-3 cement mortar according to the Chinese standard GB/T 2419-2005 [39]. Firstly, the fresh mixtures were casting into the truncated cone mold (height of 60 mm, upper inner diameter of 70 mm and bottom inner diameter of 100 mm), with the uniform ramming by two layers (Figure 1). After casting, a knife was used to remove the excessive mixtures. The truncated cone mold was lifted vertically, and then the Jump-table testing begins with the frequency of 25 times in 25 s. After completing the testing, a ruler was employed to measure the flow diameter in two vertical directions. The result was derived from the average. In particular, the fluidity testing was completed in 6 min from adding water to measure the flow diameter.

After cured for 150 and 360 d, the bulk densities were tested and averaged from six identical mortars through the testing method in the previous study [27]. In another, the porosities of samples were measured at the age of 360 d, in accordance with ASTM C642-13 (2013), and the measured method was employed in the literature [40], as Equation (1) shows:

*P* = (*M*_ssd_ − *M*_d_)/(*M*_ssd_ − *M*_w_) (1)

where *P* is the porosity (%), *M*_ssd_ means the weight in air of the saturated specimen (g), *M*_d_ means the dry weight of specimen after 24 h in the oven at 100 ± 5 °C (g) and *M*_w_ means the weight of the specimen in water (g) [40]. Moreover, the results of porosity were taken from the average of six identical mortars. To observe the shrinkage behaviors of prepared mortar, the side height values of mortars at the age of 90 and 360 d were recorded by a caliper (resolution is 0.02 mm), according to the previous study [27]. The shrinkage data are specified as the average casting height of hardening samples to the side of the adopted mold (initial casting height 40.0 mm without shrinkage).

#### 2.3.2. Determination of Long-Term Mechanical Properties

At the designed curing ages of 90, 180, 270 and 360 d, the flexural and compressive strengths were measured, using a servo-control testing machine (YAW-S, Sansi Zongheng, Shenzhen, China) with the loading capacity of 300 kN, with the accordance of GB/T 17671-1999 (ISO). According to the standard, the loading rates were, respectively, 2400 and 50 N/s for compressive and flexural strength tests [27]. The results were averaged from six identical mortar samples.

#### 2.3.3. Characterization of Acid and Sulfate Attack

**Figure 2:**
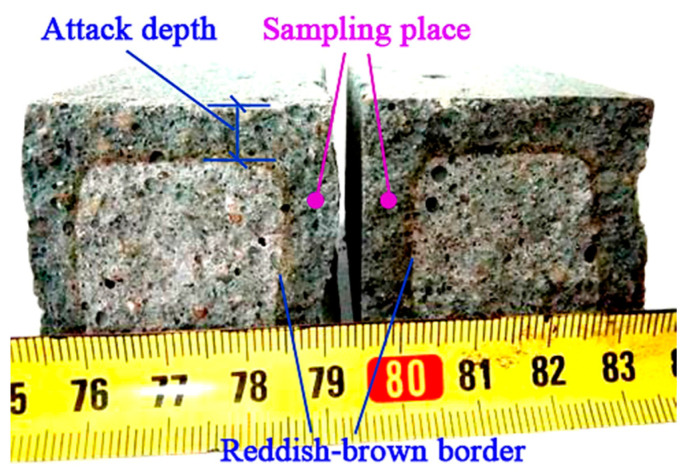
Eroded specimens taken from approximately 2 to 5 mm in depth, apart from the surface.

After analyzing the physical and mechanical properties, the representative mortars M1 and M10 (Table 3) were selected to conduct the acid and sulfate attack experiments to evaluate the resistance of SR–FFA–GEO mortars to acid and sulfate environments. The HCl and Na_2_SO_4_ attack performances were accessed by measuring the mass loss and compressive strength loss under the attack age of 28 d. The results were taken from the average with six identical samples. The attack depths were clearly presented in the cross-sections with the reddish-brown border under HCl attack because of the form of Fe(OH)_3_ at lower pH values (Figure 2). Moreover, the attack depths were recorded at an attack age of 28 d under HCl attack.

Considering the stability of geopolymer mortar, the mortars M1 and M10 cured for 360 d, at room temperature, were selected as the testing samples for chemical attack. As the controls, the mortar samples M1 and M10 were immersed into water for 28 d (the initial environmental water in the glass container measures the pH of 7.106), the mass and strength changes of which were recorded. The maximum of water-absorption rate was calculated, and the environmental pH value was also measured. Additionally, the acid and sulfate resistance tests were conducted by using the method mentioned in the previous study [26]. The geopolymer mortar samples M1 and M10 were grouped and put into pure water for 48 h, and then immersed into 5% HCl and 5% Na_2_SO_4_ solutions with a volume of 600 mL (the solution/sample volumetric ratio is 2.34) in the glass container for another 7, 14, 21 and 28 d, respectively. Here, the solutions were replaced every two days, to ensure continuous erosion under the same environments. The mass loss and strength loss of samples were recorded after chemical attack.

#### 2.3.4. Investigation into Attack Mechanisms through SEM–EDS and FTIR

After the acid and sulfate attack tests, the attack mechanisms of the SR–FFA–GEO mortar cured for 360 d are further detected, which helps to give a suggestion of durability and stability optimization. Eroded specimens of 28-day attack age were taken from approximately 2 to 5 mm in depth, apart from the outer surface (Figure 2).

The microstructures and gel compositions were detected through SEM–EDS tests. The mortar M10 with 20% SR contents, both before and after HCl and Na_2_SO_4_ attack, were investigated for the morphology and elemental components of gel products. A scanning electron microscope (SEM, Quanta FEG450, Hillsboro, OR, USA), coupled with an energy dispersive spectroscopy (EDS), was utilized [25,27,28].

Furthermore, the FTIR tests were conducted to characterize the products after chemical attack by the characteristic peak of the chemical bonds. The mortar M10 before and after HCl and Na_2_SO_4_ attack was detected. The specimens (first air-dried for 48 h and then milled) were weighed 1.3 ± 0.001 mg with 130 mg KBr pellets, to manufacture the tested specimens. A transformation infrared spectroscopy (FTIR, Nexus 8, Bruker, Karlsruhe, German) was used to detect the FTIR spectra with the wavenumber 400~3750 cm^−1^ [25,27,28].

## 3. Results and Discussion

### 3.1. Long-Term Physical Properties of SR–FFA–GEO Mortars

The fresh and physical properties of SR–FFA–GEO at 90, 150 and 360 d were shown in Table 4 and Table 5, including fluidity, bulk density, porosity and the shrinkage behavior in height.

The fluidities of the fresh geopolymer mortars were measured to elaborate the workability (Table 4). The fluidities decrease with the addition of SR from 0% to 20% (refined as SR increment of 2.2%). Moreover, the fluidity of M10 (with 20% SR) is 20.1% lower than that of M1 (without SR). This may result from the physical water absorption of SR [27], which is further evidence of the workability properties of SR–FFA–GEO materials.

Additionally, the bulk densities, mentioned in the previous research [27], were tested to further investigate the influence of SR on the density of the geopolymer mortar under long-term curing (Table 4). The bulk densities of 150 and 360 d all decrease with the increase of SR. But there are extremely small differences in bulk densities, which mainly results from the lower specific gravity of pure SR (2.35) than that of pure FFA (2.44). Moreover, the 360-day-old bulk densities are about 0.05%~0.19% lower than that of 150 days of age, which results from the moisture loss of mortars under long-term curing. This small changing trend is consistent with the previous results tested by Zhao et al. [27].

Besides, the porosities of the SR–FFA–GEO mortars at 360 d are tested with different SR contents (Table 5). The porosities increase with the addition of SR from 0% to 20%. Moreover, the porosity of M10 (with 20% SR) is 1.3 times higher than that of M1 (without SR), which demonstrates that SR addition significantly increases the porosity of SR–FFA–GEO mortar by many factors (such as chemical reaction heat, casting, vibration methods and viscosity of matrix, etc.) [27]. This is consistent with the apparent pore distributions of SR–FFA–GEO mortars presented in previous literatures [25,27].

Furthermore, the average shrinkages in casting height (initial casting height 40 mm) of the geopolymer mortars at 90 and 360 d were presented in Table 5, and the measurement method was mentioned by Zhao et al. in the previous study [27]. The average shrinkages in casting height decrease with the addition of SR from 0% to 20%. Moreover, when cured for 90 d, the M1 (without SR) shows the average casting height shrinkages of 5.20%, while the M10 (with 20% SR) has no shrinkage in casting height. In particular, there is extremely small difference in height shrinkage from 90 to 360 d, which shows that the shrinkage behavior of casting height has been stable at long-term up to 360 d. CaCO_3_ and Ca(OH)_2_ with the large surface-energy and well water-absorption, while CaSO_4_ with the expansion characteristics with water. The comprehensive effects of Ca(OH)_2_, CaCO_3_ and CaSO_4_ from SR may contribute to reducing shrinkage of FFA–GEO mortar [27].

In summary, the physical properties of bulk density, porosity and shrinkage are stable under long-term curing of up to 360 d. Therefore, SR–FFA–GEO mortar samples at 360 d-age are recommended to further investigate the resistance of geopolymer mortar to acid and sulfate attack owing to the stability of bulk density and shrinkage.

### 3.2. Long-Term Mechanical Properties of SR–FFA–GEO Mortars

The long-term mechanical properties of SR–FFA–GEO mortars were further investigated after clarifying the long-term physical properties. The compressive strengths of SR–FFA–GEO mortars were recorded as curing time of up to 360 d, as shown in Figure 2. In the previous study, Bakharev [19] found that long-term pre-curing at room temperature was beneficial for the compressive strength development of FFA–GEO materials activated by NaOH or Na_2_SiO_3_ solutions. It was revealed that the compressive strength of FFA–GEO was significantly higher if FFA–GEOs were stored for 24 h, at room temperature, before application of heat curing (75 or 95 °C), compared to that stored for 2 h [19]. Moreover, the room-temperature curing was recommended for preparing FFA–GEO mortar in the previous reference by Zhao et al. [27]. Therefore, the compressive strengths of M1 and M10 cured at room temperature were measured at 90, 180, 270 and 360 d, respectively.

**Figure 3:**
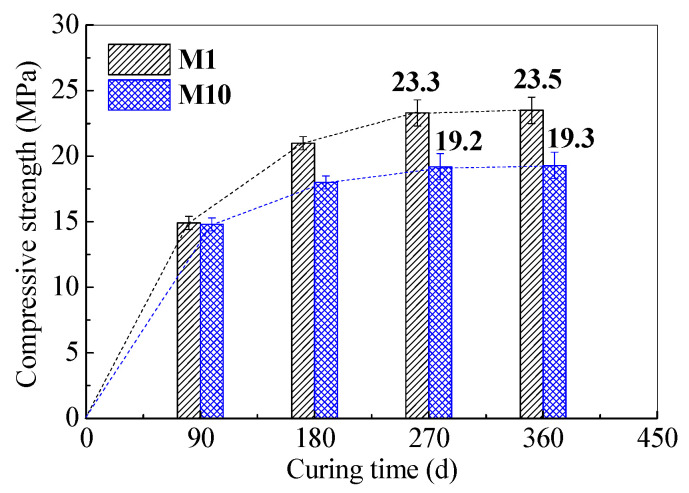
Compressive strengths development of (SR–) FFA–GEO mortars M1 and M10 at up to 360 days.

From Figure 3, it is can be seen that both of M1 and M10 show the increasing trends in compressive strengths with the curing age. However, when it exceeds 270 d, the compressive strength remains unchanged, indicating that the compressive strengths of M1 and M10 are almost stable at 23.5 and 19.3 MPa at 360 days of age, respectively. For M1, the compressive strength at 360 d is 11.9% higher than that (21 MPa) at 180 d, while the compressive strength at 360 d is 7.2% higher than that (18 MPa) at 180 d for M10. It is known that the generated gel products of N-A-S-H or (N,C)-A-S-H determine the later strength development of alkali-activated FFA–GEO materials [25,27]. Therefore, it demonstrates that the gel products in stable alkali-activated geopolymer mortars M1 and M10 do not continue to react or increase after 360 d. In general, the FFA–GEO presents the low compressive strength owing to the low activity and reaction degree of FA, as well as high porosity and heterogeneity of geopolymer materials [20,41,42]. Thus, the FFA–GEO materials cannot be directly used for structural load-bearing. However, the FFA–GEO mortar modified by SR can be used in prefabricated bricks, grouting materials, engineering soil, etc., due to about 19.3~23.5 MPa at 360 days old [43].

SR–FFA–GEO material can be used as the alternative to OPC material in civil engineering industry. However, besides the mechanical strength, the chemical resistances of OPC material are not superior due to the dissolution reaction of silicate under acid and sulfate attack [44]. Due to the inorganic calcium salts in SR, the chemical resistances of the SR–FFA–GEO mortar are unpredictable. Therefore, it needs to further investigate the chemical resistance of SR–FFA–GEO mortar to fully evaluate the comprehensive performances.

### 3.3. Strength and Mass Changes of SR–FFA–GEO Mortar under Water Environment

To clarify the influence of water environment on the stability of SR–FFA–GEO mortar, the compressive strength and mass of M1 and M10 cured for 360 d were recorded under the water environment for 28-day immersion age (Table 6). During the testing, the masses of M1 and M10 increase first after being immersed in water and then reach the maximum after 7 h, which is mainly caused by water-absorption rate of mortars. The maximum water-absorption rate of M1 is 1.5%, while that of M10 measures 1.8% which is close to that of M1. Compared with water-absorption rate 7.11% of the cement mortar and 6.07% of the geopolymer mortar (synthesized using fly ash/kaolin activated by the mixtures of NaOH and Na_2_SiO_3_ solutions) referred in the literature [37], the water-absorption rate of M1 and M10 are lower. Here, the three-dimension network structure, the high cementation as well as the discontinuity of pores of (soda residue–) fly-ash-based geopolymer may contribute to the resist penetration of water into the matrix, which leads to the lower water absorption for M1 and M10. As the immersion age increases, the pH value of environmental water reaches 11.345 (the original pH is 7.106) for M1, and while the pH value of environmental water measures 11.248 for M10 (Table 6). Both of the alkaline environments are caused by the remaining NaOH released into the environment. At the same time, there is little difference in the masses of FFA–GEO or SR–FFA–GEO mortars as the immersion-age up to 28 d under a water environment. In particular, the compressive strengths of M1 and M10 remain unchanged under immersion.

Therefore, the mortars cured for 360 d have no significant changes in compressive strength and mass losses under a water environment. Moreover, the M1 and M10 cured for 360 d are recommended to further analyze the influence of HCl and Na_2_SO_4_ environment on the performances of the SR–FFA–GEO mortars, with the exclusion of the continuation of internal hydration reaction.

### 3.4. Strength and Mass Losses of SR–FFA–GEO Mortar under HCl and Na_2_SO_4_ Attack

**Figure 4:**
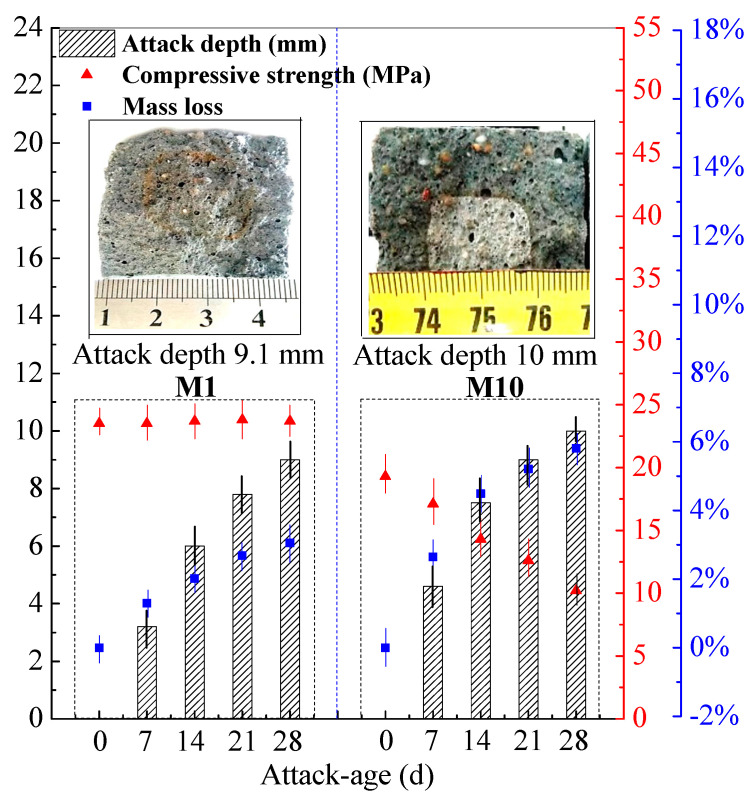
Compressive strength, mass loss and attack depth of M1 and M10 (cured for 360 d) under HCl-attack for 28 d.

The hydrochloric acid (HCl) attack test was performed on M1 and M10 at 360 d. Under 5% HCl attack, the surfaces of M1 and M10 mortar slowly produce some bubbles, which may be attributed to the CO_2_ from the contact of CaCO_3_ in SR and HCl. From the attack depth by the reddish-brown border (Figure 2 in the aforementioned section), the middle cross-section of M10 presents 10.0 mm in attack depth under a 28-day attack age, which is higher than the attack depth of M1 (measures 9.0 mm), as shown in Figure 4.

The compressive strength and mass loss under a 28-day attack age were recorded in Figure 4. The mass losses of M1 and M10 increase with the attack age. The mass loss of M10 reaches 5.82% at a 2-day attack age, while the mass loss of M1 reaches 3.06%. However, the compressive strength of M1 keeps stable, and that of M10 decreases by 47.2% after a 28-day attack age. The mortar M1 possesses the good resistance to HCl attack, owing to a three-dimensional stable network structure. Thus, M1 has little mass loss (3.06%) and unchanged compressive strength under HCl attack, which is consistent with that of high-strength FA–GEO material mentioned in the previous study [34,45]. Moreover, the mortar M10 possesses the worse resistance to HCl attack than that of M1, which may result from the calcites in SR. The formed M10 was porous due to a variety of factors, such as pouring and vibration, viscosity, reaction heat, etc. [27]. Here, the calcites in porous SR reacted with HCl, to produce more porosity for M10, which led to the decrement of compressive strength. However, the resistance of M10 to HCl attack (mass loss 5.82% and strength loss 47.2%) is higher than that of OPC mortar in the previous study (mass loss 40.97% and strength loss 85.2%) [37] at a 28-day attack age (Table 7).

**Figure 5:**
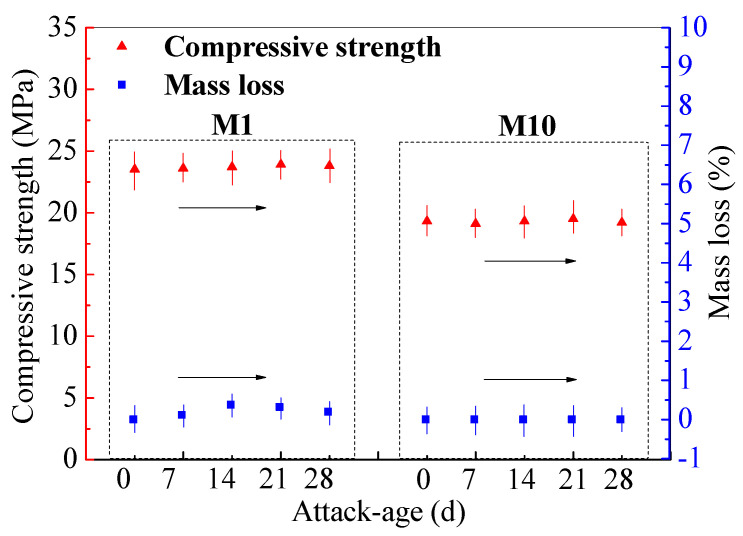
Compressive strength and mass loss of M1 and M10 (cured for 360 d), under Na_2_SO_4_-attack for 28 d.

The sodium sulfate (Na_2_SO_4_) attack test was performed on M1 and M10 cured for 360 d at room temperature. Under 5% Na_2_SO_4_ solution, the compressive strength and mass loss were recorded for 28-day attack age (Figure 5). It can be seen that no mass losses occur for M1 and M10, while the compressive strengths of M1 and M10 remain stable with the attack age.

In the previous study, the FA activated by sodium silicate solution (modulus 1.2, and mass concentration 32%) was prepared geopolymer cured for 1 d at 25 °C and then for 2 d at 80 °C. The compressive strength of the prepared FA–GEO increases with the attack age under 5% Na_2_SO_4_ attack [46]. The compressive strength of the mentioned FA–GEO cannot reach the maximum at temperature-curing for 3 d. Under the Na_2_SO_4_ attack, the geopolymerization reaction still continues to occur, which may be because the environmental Na^+^ cations enter the network structure formed with Si-O-Al chains through diffusion and promote the positive effect on the polymerization degree of Si-O-Al bond [34,45]. Therefore, without compressive strength loss and mass loss occurring, it can be determined that the resistances of M1 and M10 cured for 360 d to Na_2_SO_4_ attack are superior and evenly superior to that of OPC mortar (Table 7). That is because that the calcium silicate hydrated gels in OPC mortar are continuously dissolved by HCl, and the sands fall off from the mortar under 5% HCl attack, which leads to the higher losses in mass and compressive strength for OPC mortar.

### 3.5. Microstructures and Gel Products under HCl and Na_2_SO_4_ Attack through SEM–EDS Analysis

Compressive strengths and mass losses are the macroscopic characterization of the chemical attack phenomenon, and it is necessary to further characterize the changes before and after chemical attack from the microstructure and gel product. It helps to reveal the attack mechanism of HCl and Na_2_SO_4_ solution.

**Figure 6:**
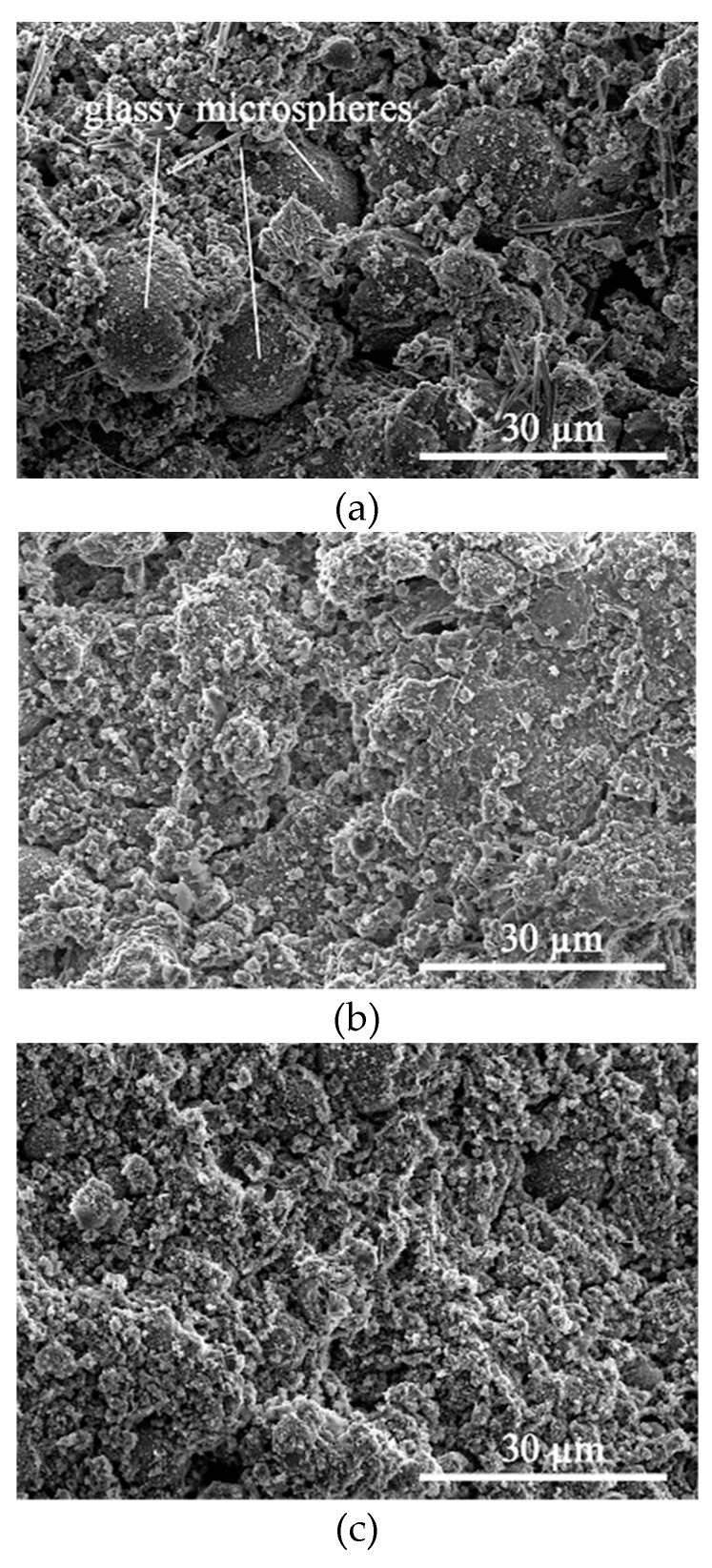
SEM images of M10: (**a**) before attack, (**b**) HCl-attack for 28 d and (**c**) Na_2_SO_4_-attack for 28 d.

**Figure 7:**
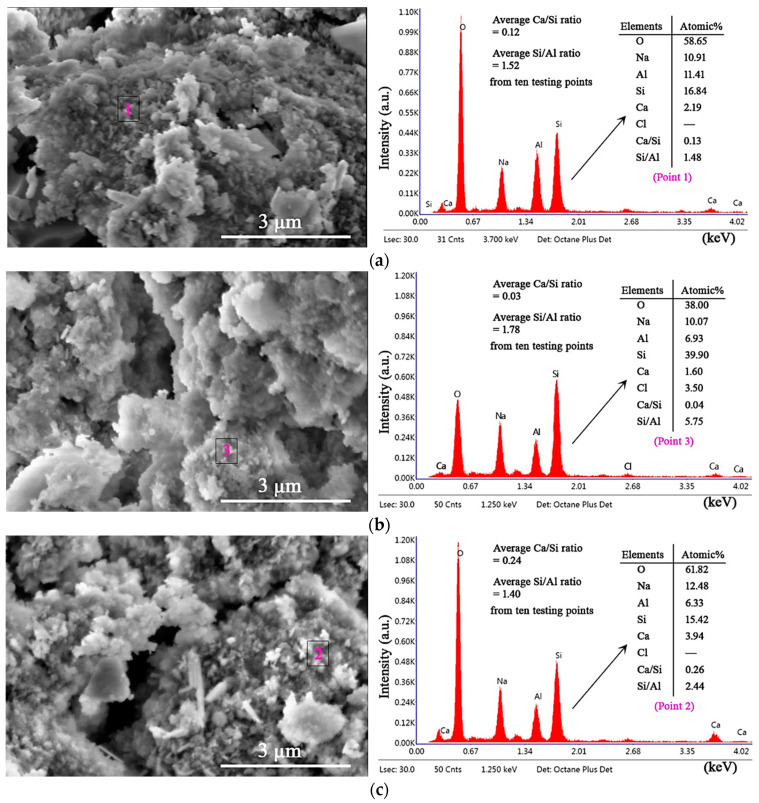
EDS spectra of representative SEM micro-areas for M10: (**a**) before attack, (**b**) HCl-attack for 28 d and (**c**) Na_2_SO_4_-attack for 28 d.

After clarifying the compressive strength and mass loss of SR–FFA–GEO mortars under HCl and Na_2_SO_4_ environment, the attack mechanism of SR–FFA–GEO mortar was further revealed through the analysis of microstructures and elemental composites before and after attack. The ten different locations were probed when determining the Ca/Si and Si/Al ratios from the EDS. The SEM images are the representative images in the revised manuscript (Figure 6 and Figure 7). The testing method of SEM–EDS is to first probe the entire window to select the specific gel product not standard stand according to the elemental components (Si, Al, Na, O, Ca, etc.) [26]. Then, the selected representative micro-areas were used to probe the specific locations to get effective information of gel products before and after chemical attack. The testing results are obtained from the elemental components at the representative micro-areas. The abovementioned method greatly removes the influence of standard sand (quartz), and that pays more attention to the change of gel products.

The morphologies of M10 were observed by SEM images before and after attack (Figure 6). There still are some undissolved glassy microspheres of FA in mortar M10 before HCl and Na_2_SO_4_ attack. After HCl attack for 28 d, the glassy microspheres are further dissolved, whereas the glassy microspheres have no change after Na_2_SO_4_ attack for 28 d. After magnification, it is found that the formed gel products are observed on the surface of M10 (Figure 7a–c).

From the representative EDS spectra and elemental components from area-scanning technology (Figure 7), calcium elements (Ca) in gel products of M10 decrease after HCl attack, while there are no sulfate elements (S) in gel products after Na_2_SO_4_ attack. Under Na_2_SO_4_ attack, SO_4_^2−^ ions in environment cannot enter the geopolymer mortar through diffusion, and also average Ca/Si ratios increase from 0.12 to 0.24 and average Si/Al ratios decrease from 0.52 to 1.40 in gel products, which illustrates that environmental Na_2_SO_4_ promotes the absorption of Na^+^ from environment and Ca^2+^ into geopolymer network structure, as well as evenly improves the geopolymeric degree of Si-O-Si bonds due to the lower Si/Al ratio in gel products. In particular, the Si/Al ratio (point 3, Figure 7b) increases five-fold, owing to detecting some unreactive fly ash particles or some quartz, which leads to dramatical increments of Si contents. Therefore, the changes of Ca, Si and Al are more verified by the average values from different locations to illustrate the results.

Emphatically, from the experimental results, the fluidity, porosity and shrinkage of SR–FFA–GEO mortar are influenced more by the addition percentage of SR (Table 4 and Table 5). Moreover, after physical properties testing, the long-term compressive strengths are focused on for M1 without SR and M10 with 20% SR. Therefore, the compressive strengths before and after chemical attack are still influenced more by the addition percentage of SR, and even the attack mechanisms of the HCl solution are derived from the addition of SR.

### 3.6. Chemical Bonds of Gel Products under HCl and Na_2_SO_4_ Attack through FTIR Analysis

**Figure 8:**
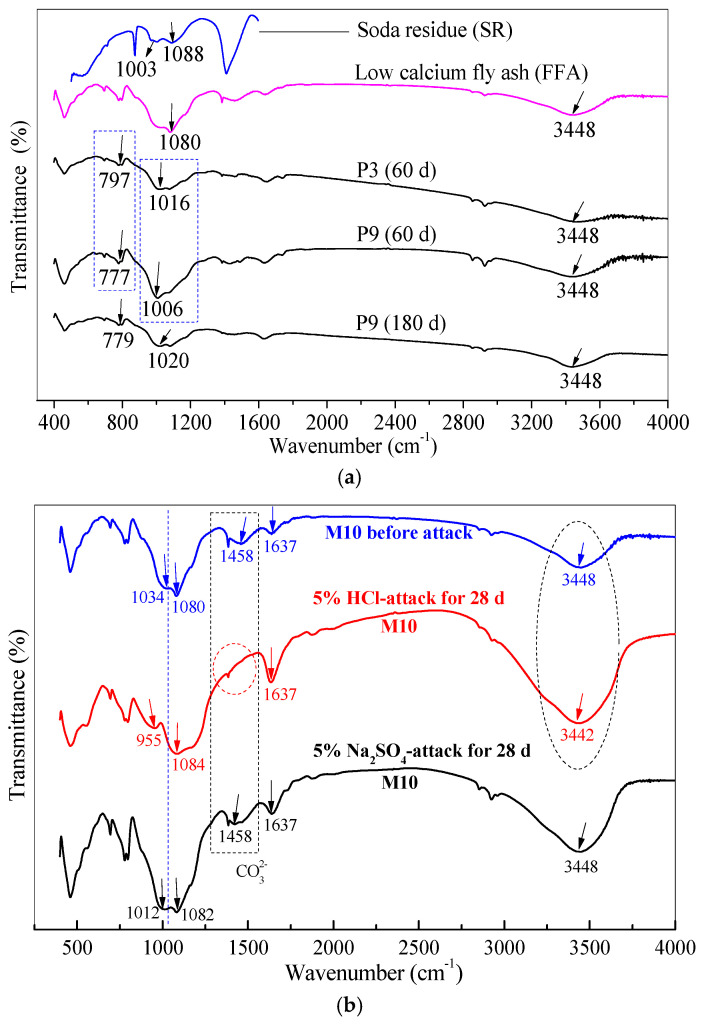
(**a**) FTIR spectra of low-calcium fly ash (FFA), soda residue (SR), paste P3 and paste P9 cured at room temperature for 60 and 180 d; (**b**) FTIR spectra of SR–FFA–GEO mortar M10 cured for 360 d before and after 5% HCl and 5% Na_2_SO_4_ attack.

The FTIR tests were employed to further analyze the changes in the chemical bonds of SR–FFA–GEO mortar and clarify the attack mechanism under HCl and Na_2_SO_4_ environment. The measured FTIR spectra of SR, FFA, paste P3 without SR and paste P9 with 20% SR were presented for 180-day-old samples in the previous study (Figure 8a). The measured FTIR spectra of M10 with 20% SR (cured for 360 d) before and after HCl and Na_2_SO_4_ attack are presented in Figure 8b.

All wavenumbers at 777, 797 and 779 cm^−1^ correspond to the bending vibration absorptions of Si-O and Al-O bonds. The main absorption peaks attributed to Si-O-T (Si or Al) with the asymmetrical stretching vibrations are at the region from 1000 to 1100 cm^−1^ [15,47,48]. The absorptions at 1458 cm^−1^ mainly are attributed to the asymmetric stretching vibration of CO_3_^2−^ bond in carbonate, and then the absorption peaks at 1637 and 3448 cm^−1^, respectively, correspond to the bending vibration of H-O-H bond and the stretching vibration of -OH bonds in weakly bound water [19,45].

In the previous study, the wavenumber at 1080 cm^−1^ in FFA shifts to the lower wavenumber at 1016 cm^−1^ in paste P3 (FFA–GEO paste). When 20% SR are added into paste P3 to become paste P9 (SR–FFA–GEO paste), the band of Si-O-T (Si or Al) with the asymmetrical stretching vibration shifts to the lower wavenumber (1016 cm^−1^→1006 cm^−1^) (Figure 8a). The peak of Si-O and Al-O bonds with the bending vibrations also shifts to a lower wavenumber (797 cm^−1^→777 cm^−1^), which illustrates that the 20% SR additives have promoted the geopolymerization of Si-O-Al chains in pastes for 60 d. However, for paste P9, the absorption peak of Si-O-T (Si or Al) bond with the asymmetrical stretching vibration shifts to the higher wavenumber (1006 cm^−1^→1020 cm^−1^). This shows that the polymerization of Si-O-Si bond distinctly occurs and further promotes the formation of C-S-H gels by the 20% SR additives during 60~180 d. Additionally, the alkaline SRs consist of the high contents of Ca(OH)_2_, CaCO_3_, CaCl_2_ and CaSO_4_. The Ca^2+^ cations dissolved from the additional SRs participate in the form of C-S-H and C-A-S-H gels by providing the nucleation location for (N,C)-A-S-H or/and N-A-S-H [29]. Therefore, the gel products of (N,C)-A-S-H, C-S-H and C-A-S-H are verified in SR–FFA–GEO (paste and mortar) with 20% SR.

The SR was used with blend of FFA and alkali-activator solution to prepare the geopolymer grouting paste for goaf backfill [25]. The prepared paste occurs the chemical reaction of geopolymerization between Si-O and Al-O bonds to generate the mixtures of (Ca, Na)-containing aluminosilicate polymer gel (N,C)-A-S-H and calcium silicate hydrated gel C-S-H. Additionally, the SR also was used with FFA, beta-hemihydrate gypsum and alkali-activator solution, to manufacture the geopolymer slurry for goaf backfill [49]. The reaction mechanism of geopolymerization and hydration process still happen to form the coexistence of (N,C)-A-S-H and C-S-H gels, along with the hydration of gypsum. These above-verified gel products support the chemical reaction mechanism and hardening mechanism of SR–FFA–GEO mortar M10 for the FTIR results.

Under 5% HCl attack, for the M10 with 20% SR, the weak absorption peak at 1034 cm^−1^ shifts to a lower wavenumber at 955 cm^−1^ (Figure 8b), indicating that HCl dissolves the glassy microspheres of FFA to generate the silicon and aluminum monomers. Moreover, the absorption peak attributed to CO_3_^2−^ bond disappeared at 1458 cm^−1^. The peak intensities at 1637 and 3448 cm^−1^ increase, and also the absorption peak at 3448 cm^−1^ shifts to the lower wavenumber 3442 cm^−1^, which indicates that the carbonates (namely calcite) from SR decompose under HCl attack, and a part of water is adsorbed on the surface of geopolymer. In the previous study, Zhao et al. [26] found through FTIR and XRD that calcites were wrapped with N-A-S-H and (N,C)-A-S-H gels in SR–FFA–GEO paste, and calcites did not occur chemical reaction. But here, with HCl solution attack, the environmental HCl solution goes gently inside the formed gels and reacts with the calcites to increase the porosity and decrease the compressive strength.

Under 5% Na_2_SO_4_ attack, the absorption peak at 1034 cm^−1^ (before attack) shifts to the lower wavenumber 1012 cm^−1^ (Figure 8b), which shows that Na_2_SO_4_ promotes the geopolymerization of Si-O-Al bonds, and more additional Al (dissolved from fly ash particles) promote the absorption of Na^+^ and Ca^2+^ cations in network structure. The higher geopolymeric degrees of Si-O-Al bonds determine the lower Si/Al molar ratio in the gel product. The aluminosilicate gel is formed by Si-O-Al or Si-O-Si bonds, owing to the charge balance between Al and metal cations, and the positive charge is compensated by metal cation (Na^+^, K^+^ and Ca^2+^). Therefore, the higher Si/Al ratios, which are reflected in Figure 8b, determine the more dissolution of Al and resist penetration of SO_4_^2−^ ions. These conclusions can be clarified by SEM–EDS tests. Moreover, the more environmental Na^+^ cations entering the Si-O-Al network structure were also verified by the SEM–EDS tests under Na_2_SO_4_ attack.

## 4. Conclusions

The aim of this paper is to validate the resistance of soda residue/low-calcium-fly-ash-based geopolymer mortar to acid and sulfate environments and explore the chemical attack mechanism of the geopolymer mortar. Conclusions could be obtained as follows:

(1) The compressive strengths (19.3 MPa) of soda residue/low-calcium-fly-ash-based geopolymer mortar with 20% soda residue are stable at 360 days old, when cured at room temperature. In particular, the compressive strength, porosity and shrinkage are influenced more by the addition percentage of soda residue. Moreover, the geopolymer mortar with 20% soda residue (with low water absorption of 1.8%) keeps stable in compressive strength and mass loss under water environment for 360 d.
(2) The soda residue/low-calcium-fly-ash-based geopolymer mortar with 20% soda residue cured for 360 d at room temperature is recommended to investigate the resistance to acid and sulfate attack owing to the better stability with the exclusion of internal hydration.
(3) Under 5% HCl solution attack for 28 d, the mass loss of the geopolymer mortar with 20% soda residue reaches 5.82%, and the compressive strength loss is 47.2%. However, under 5% Na_2_SO_4_ solution attack for 28 d, there is no compressive strength and mass losses for the geopolymer mortar with 20% soda residue. Therefore, the geopolymer mortar with 20% soda residue possesses the superior resistance to Na_2_SO_4_ attack, as well as the better resistance to HCl attack than that of ordinary Portland cement material.
(4) From the SEM–EDS and FTIR analysis, the calcites from soda residue cause the chemical reaction with the environmental HCl to produce some CO_2_ gas, which leads to the losses in compressive strength and mass under HCl attack. Thus, the attack mechanisms of HCl solution are derived from the addition of soda residue. Moreover, more Na^+^ cations entering the Si-O-Al structure make soda residue/low-calcium-fly-ash-based geopolymer mortar obtain the superior resistance to Na_2_SO_4_ attack without compressive strength loss and mass loss.

In summary, the resistances of the soda residue/low-calcium-fly-ash-based geopolymer mortar (4:1 mass ratio of low calcium fly ash and soda residue, 1:3 of cement–sand, 8 mol/L NaOH solution and 0.75 liquid–solid ratio) to 5% HCl and 5% Na_2_SO_4_ environments are superior to that of ordinary Portland cement mortar. The contribution of this work is to validate the resistance performance to acid and sulfate environments and reveal the attack mechanism of the prepared geopolymer mortar incorporating soda residue. It supports the durable evaluation of soda residue/low-calcium-fly-ash-based geopolymer mortar. However, the soda residue/low-calcium-fly-ash-based geopolymer mortar presents the high porosity of 14.36% in the physical properties. In the next works, the porosity-reduction method of low-calcium-fly-ash-based geopolymer mortar incorporating soda residue will be further explored, to achieve high strength or evaluate the freezing stability.

## Figures and Tables

**Table 1:** Chemical compositions of solid powders by XRF.

Materials	Chemical Composites and Mass Percentages ^1^							
Soda residue (SR)	CaCO_3_	Ca(OH)_2_	CaCl_2_	CaSO_4_	NaCl	SiO_2_	Al_2_O_3_	Acid Insoluble
Percentage (%)	64.0	10.0	6.0	3.0	4.0	3.0	2.0	8.0
Low-calcium fly ash (FFA)	SiO_2_	Al_2_O_3_	Fe_2_O_3_	CaO	FeO	MgO	LOI ^2^	Others
Percentage (%)	51.20	25.32	7.80	5.32	2.20	1.80	3.05	3.31

^1^ Data from the literature by Zhao et al. [27]. ^2^ LOI refers to the loss on ignition at the temperature of 1000 °C (%).

**Table 2:** Physical indexes of raw material SR and FFA.

Solid Powders	Specific Surface Area (m^2^/kg)	pH Value at *w*_100_^1^	Amount Passing #325 Sieve	Mean Particle Size (mm)	Specific Gravity ^2^
SR	—	8.35	24%	0.25	2.35
FFA	510	5.90	76%	—	2.44

^1^*w*_100_ refers to the measured pH at 100% of water content under room temperature. ^2^ Data from the literature by Zhao et al. [27].

**Table 3:** Mixing proportions of the geopolymer mortars prepared at room temperature. Liquid–solid ratio is 0.75, and cement–sand ratio is 1:3.

No.	FFA (g)	SR (g)	SR Content	Standard Stand (g)	NaOH Solution (mol/L)	Na/Si Ratio	Al/Si Ratio	Ca/Si Ratio
M 1	450	0	0.0%	1350	8	0.54	0.57	0.11
M 2	440	10	2.2%	1350	8	0.55	0.57	0.12
M 3	430	20	4.4%	1350	8	0.57	0.57	0.12
M 4	420	30	6.7%	1350	8	0.58	0.57	0.13
M 5	410	40	8.9%	1350	8	0.59	0.57	0.13
M 6	400	50	11.1%	1350	8	0.61	0.57	0.14
M 7	390	60	13.3%	1350	8	0.63	0.57	0.15
M 8	380	70	15.6%	1350	8	0.64	0.57	0.15
M 9	370	80	17.8%	1350	8	0.66	0.57	0.16
M10	360	90	20.0%	1350	8	0.68	0.57	0.17

Note: Na/Si ratio refers to the molar ratio of Na from NaOH to Si from SiO_2_. Al/Si ratio refers to the molar ratio of Al from Al_2_O_3_ to Si from SiO_2_. Ca/Si ratio refers to the molar ratio of Ca from CaCl_2_, CaSO_4_, CaO and Ca(OH)_2_ to Si from SiO_2_.

**Table 4:** The fluidity and bulk density of the geopolymer mortars with different SR contents cured for 150 and 360 d, at room temperature.

No.	SR Content	Fluidity (mm)	Bulk Density (g/cm^3^)				Difference Percentage for 150~360 d
			150 d ^1^	Difference	360 d	Difference	
M 1	0.0%	184	2.145	±0.017	2.143	±0.015	0.09%
M 2	2.2%	181	2.135	±0.015	2.134	±0.013	0.05%
M 3	4.4%	176	2.123	±0.023	2.12	±0.020	0.14%
M 4	6.7%	171	2.117	±0.016	2.115	±0.014	0.09%
M 5	8.9%	164	2.113	±0.020	2.111	±0.022	0.09%
M 6	11.1%	158	2.107	±0.015	2.104	±0.017	0.14%
M 7	13.3%	153	2.104	±0.025	2.101	±0.023	0.14%
M 8	15.6%	152	2.101	±0.012	2.097	±0.012	0.19%
M 9	17.8%	149	2.097	±0.014	2.093	±0.013	0.19%
M10	20.0%	147	2.093	±0.010	2.091	±0.012	0.10%

^1^ Part of data from the literature by Zhao et al. [27].

**Table 5:** The porosity and shrinkage of the SR–FFA–GEO mortars with different SR contents cured for 90 and 360 d at room temperature.

No.	SR Content	360 d Porosity	Initial Casting Height (mm)	Final Casting Height (mm)	Average Shrinkage for 90 d	Initial Casting Height (mm)	Final Casting Height (mm)	Average Shrinkage for 360 d
M 1	0.0%	6.23%	40.00	37.92	−5.20%	40.00	37.92	−5.19%
M 3	4.4%	8.34%	40.00	38.42	−3.96%	40.00	38.42	−3.97%
M 5	8.9%	10.34%	40.00	38.98	−2.56%	40.00	38.98	−2.56%
M 7	13.3%	11.67%	40.00	39.54	−1.15%	40.00	39.56	−1.09%
M 9	17.8%	13.14%	40.00	40.00	0.00%	40.00	40.00	0.00%
M10	20.0%	14.36%	40.00	40.00	0.00%	40.00	40.00	0.00%

**Table 6:** The compressive strengths and masses of M1 and M10 (cured for 360 d), under a water environment, for a 28-day immersion age.

No.	SR Content	Maximal Water Absorption at 7 h	Environmental pH Value	Immersion Age	Compressive Strength (MPa)	Mortar Mass (g)
M 1	0.0%	1.5%	11.345	0 d	23.5	543
				7 d	23.6	541
				14 d	23.6	540
				21 d	23.7	540
				28 d	23.7	540
M10	20.0%	1.8%	11.248	0 d	19.3	540
				7 d	19.3	542
				14 d	19.4	541
				21 d	19.5	541
				28 d	19.5	541

**Table 7:** The compressive strength and mass losses of M10 (cured for 360 d) and Ordinary Portland Cement (OPC) mortar under HCl-attack and Na_2_SO_4_-attack environment for 28 d.

Samples	SR Content	5% HCl-Attack		5% Na_2_SO_4_-Attack	
		Strength Loss	Mass Loss	Strength Loss	Mass Loss
M10	20%	47.2%	5.82%	0.0%	0.00%
OPC ^1^	—	85.2%	40.97%	5.8%	6.83%

^1^ Part of data from the literature [37].

## Data Availability

The data presented in this study are available on request from the corresponding author. The data are not publicly available due to the privacy restrictions.
